# Machine learning approach to estimate soil matric potential in the plant root zone based on remote sensing data

**DOI:** 10.3389/fpls.2022.931491

**Published:** 2022-08-15

**Authors:** Rodrigo Filev Maia, Carlos Ballester Lurbe, John Hornbuckle

**Affiliations:** Centre for Regional and Rural Futures, Deakin University, Hanwood, NSW, Australia

**Keywords:** machine learning, remote sensing, soil matric potential, NDVI, evapotranspiration, irrigation

## Abstract

There is an increasing interest in using the Internet of Things (IoT) in the agriculture sector to acquire soil- and crop-related parameters that provide helpful information to manage farms more efficiently. One example of this technology is using IoT soil moisture sensors for scheduling irrigation. Soil moisture sensors are usually deployed in nodes. A more significant number of sensors/nodes is recommended in larger fields, such as those found in broadacre agriculture, to better account for soil heterogeneity. However, this comes at a higher and often limiting cost for farmers (purchase, labour costs from installation and removal, and maintenance). Methodologies that enable maintaining the monitoring capability/intensity with a reduced number of in-field sensors would be valuable for the sector and of great interest. In this study, sensor data analysis conducted across two irrigation seasons in three cotton fields from two cotton-growing areas of Australia, identified a relationship between soil matric potential and cumulative satellite-derived crop evapotranspiration (ET_cn_) between irrigation events. A second-degree function represents this relationship, which is affected by the crop development stage, rainfall, irrigation events and the transition between saturated and non-saturated soil. Two machine learning models [a Dense Multilayer Perceptron (DMP) and Support Vector Regression (SVR) algorithms] were studied to explore these second-degree function properties and assess whether the models were capable of learning the pattern of the soil matric potential-ET_cn_ relation to estimate soil moisture from satellite-derived ET_c_ measurements. The algorithms performance evaluation in predicting soil matric potential applied the k-fold method in each farm individually and combining data from all fields and seasons. The latter approach made it possible to avoid the influence of farm consultants’ decisions regarding when to irrigate the crop in the training process. Both algorithms accurately estimated soil matric potential for individual (up to 90% of predicted values within ±10 kPa) and combined datasets (73% of predicted values within ±10 kPa). The technique presented here can accurately monitor soil matric potential in the root zone of cotton plants with reduced in-field sensor equipment and offers promising applications for its use in irrigation-decision systems.

## Introduction

Population growth in recent decades has boosted water demand worldwide, but water use and water consumption trends at current rates are unsustainable (FAO, 2017). Therefore, multidisciplinary research efforts in water management are required to achieve a sustainable future (Cosgrove and Loucks, 2015). This includes research on water management in agriculture, which accounts for ~70% of the freshwater use worldwide and is the most water-demanding of all economic sectors (Pimentel et al., 2004). Irrigation is the activity that requires most of the water resources available and influences a variety of biophysical processes in plants that directly relate to yield.

Optimizing irrigation scheduling decisions in agriculture is a challenge that needs to be met to manage water resources more efficiently and improve crop water productivity (Jägermeyr, 2020; Chaudhary and Srivastava, 2021). Technology development is critical in providing more accurate tools for monitoring soil and crop water status at different scales (Bittelli, 2011; Cahn and Johnson, 2017; Saad and Gamatié, 2020). A range of approaches are available for direct and indirect measurements of the soil and plant water status (see Jones (2006)). For irrigation scheduling purposes, soil moisture monitoring has traditionally been the methodology used, and it is generally preferred to other methods due to its suitability for irrigation automation. An evaluation of available soil moisture measurement technologies and their limitations can be found in Susha Lekshmi et al. (2014). Water content sensors provide helpful information to determine when to irrigate, but soil texture influences the measurements requiring site-specific calibration (Cahn and Johnson, 2017). Soil matric potential sensors indicate how readily water is accessible for plants (Jones, 2006), do not require soil-specific calibration and are generally preferred for water management in vegetable crops (Thompson et al., 2007; Cahn and Johnson, 2017).

One of the limitations to the wide use of soil matric potential sensors at the commercial scale is that they only provide point-source measurements, and thus, many sensors are needed in large-scale broadacre farming to accounting for soil spatial variability (Cahn and Johnson, 2017). To monitor large heterogeneous farms with sensors, Wireless Sensor Networks can be used to interconnect them and make data available online in near real-time. However, it considerably increases the cost of the monitoring system (acquisition, installation, maintenance, among others), which has been reported as one of the significant barriers to the wide adoption of these technologies by farmers (Blasch et al., 2022). Remote sensing techniques can estimate soil moisture and allow capturing the existing spatial variability in large areas. However, these techniques still need improvement and are not accurate enough to directly estimate soil moisture at a field scale suitable for irrigation scheduling (Shunlin Liang, 2020). Therefore, methodologies that could minimize the number of in-field sensors without losing soil moisture monitoring capability/intensity would greatly value water managers.

While not yet ready to accurately directly monitor soil moisture at the field scale for irrigation scheduling, remote sensing-based approaches are helpful for the estimation of crop evapotranspiration through vegetation indices gathered from satellite, airborne and drone-based platforms following the FAO56 method (Pereira et al., 2015; Pôças et al., 2020). In this approach, crop evapotranspiration (ET_c_) is estimated by multiplying the reference evapotranspiration (ET_o_) obtained from data collected at nearby weather stations by a site-specific crop coefficient that is obtained from vegetation indices that can be monitored at high temporal and spatial resolution. The Normalized Difference Vegetation Index (NDVI) is the most widespread vegetation index and is linearly related to the crop coefficient (Trout and Johnson, 2007). This ET_c_/NDVI approach enables water managers to monitor crop water requirements at individual sites throughout the growing season. However, the rate at which moisture is depleted from the soil is related to the crop evapotranspiration, which varies with the crop phenological stage, and thus, their relationship is time dependent. This time-dependent relationship hinders the possibility of estimating or accurately predicting soil matric potential from evapotranspiration measurements by conventional data processing techniques. In this work, we studied this relationship when soil matric potential is used to monitor soil moisture in gravity surface irrigated systems. The hypothesis was that machine learning models can learn the interaction between soil-, crop-, and weather-related parameters to estimate soil matric potential in the root zone from remotely sensed evapotranspiration measurements.

With the adoption of Information and Communication Technology in agriculture and the substantial volume of data generated, data-driven machine learning techniques that can organize data from different sources and the power to learn from them become essential (Alzubi et al., 2018; Benos et al., 2021). Machine learning techniques have been applied in agriculture in various farming practices, with those related to crop management activities (disease detection, yield prediction, among others) receiving most of the attention. Although growers see potential in using the Internet of Things, remote sensing, and machine learning (Agriculture 4.0) for having better decision-making processes, particularly in irrigation, substantially less work has been undertaken on water management activities (Benos et al., 2021). Within the studies focused on water management, machine learning techniques have been applied to estimate groundwater reservoirs, soil moisture (Paloscia et al., 2013; Coopersmith et al., 2016; Prasad et al., 2018; Singh et al., 2019; Babaeian et al., 2021; Greifeneder et al., 2021; Grillakis et al., 2021; Orth, 2021; Sungmin and Rene, 2021), evapotranspiration (Ponraj and Vigneswaran, 2020), and provide irrigation control (González-Briones et al., 2019; Kondaveti et al., 2019; Murthy et al., 2019; Akshay and Ramesh, 2020; Campoverde et al., 2021; Ikidid et al., 2021; Perea et al., 2021; Bhoi et al., 2021a), among other applications (Liakos et al., 2018; Cardoso et al., 2020; Perea et al., 2021; Bhoi et al., 2021b). The machine learning techniques applied in these studies are shown in Table 1, following the classification suggested in (Liakos et al., 2018) and considering two additional categories: Multi-Agent System (MAS) and Genetic Algorithm. The algorithms applied to estimate soil moisture are Bayesian models, Artificial Neural Networks (ANN), Regression models, Decision Tree models and MAS. Several research papers rely on neural network algorithms to classify or estimate crop parameters. Support Vector Regression (SVR, also called SVM in most papers as shown in Table 1) and Decision Tree-based algorithms are also primarily used for the same purposes.

**Table 1:** Algorithms used for a range of applications in agriculture using remote sensing (R) and sensors data (S).

Application	BM	SVM	ANN	Regression	DT	MAS	Genetic
Water estimation	–	S	S	S	–	–	–
Soil moisture	R, S	–	R, S	R, S	R, S	S	–
Evapotranspiration	–	–	R, S	S	S	–	R, S
Irrigation control	S	S	S	–	S	–	–
Rainfall prediction	–	S	–	–	–	–	–
Total	4	4	9	5	9	2	1

BM, Bayesian models; SVM, support vector machine; ANN, artificial neural network; DT, decision tree; MAS, multi-agent system.

This work proposes the original approach of using the relationship between soil matric potential and the cumulative evapotranspiration between irrigation events expressed in kPa/mm aiming to (i) explore the proposed relation over the cotton growing season and (ii) assess the feasibility of estimating soil matric potential in the cotton root zone (0.20 m below ground) from remotely sensed evapotranspiration by using machine learning models. The kPa/mm relation may represent the dynamics of crop water use during the season. Supported vector models and ANN were the machine learning models applied because of their capability to process time-dependent parameters. The models’ performance in estimating soil matric potential at bay level was evaluated and compared following two approaches: (i) when models were trained with data for each farm and growing season, and (ii) when models were trained with data from all the sites and seasons combined. The second approach was implemented to avoid any influence water managers’ decision practices could have on the algorithm responses.

The study contributes to the research on implementing machine learning techniques in irrigation water management that are scarce in the literature compared to crop management activities. It presents an approach to cotton producers of the main cotton-growing areas of Australia that would allow them to monitor soil matric potential with a reduced number of in-field sensors and potentially optimize on-farm water management in these systems.

## Materials and methods

### Site locations and characteristics

**Figure 1:**
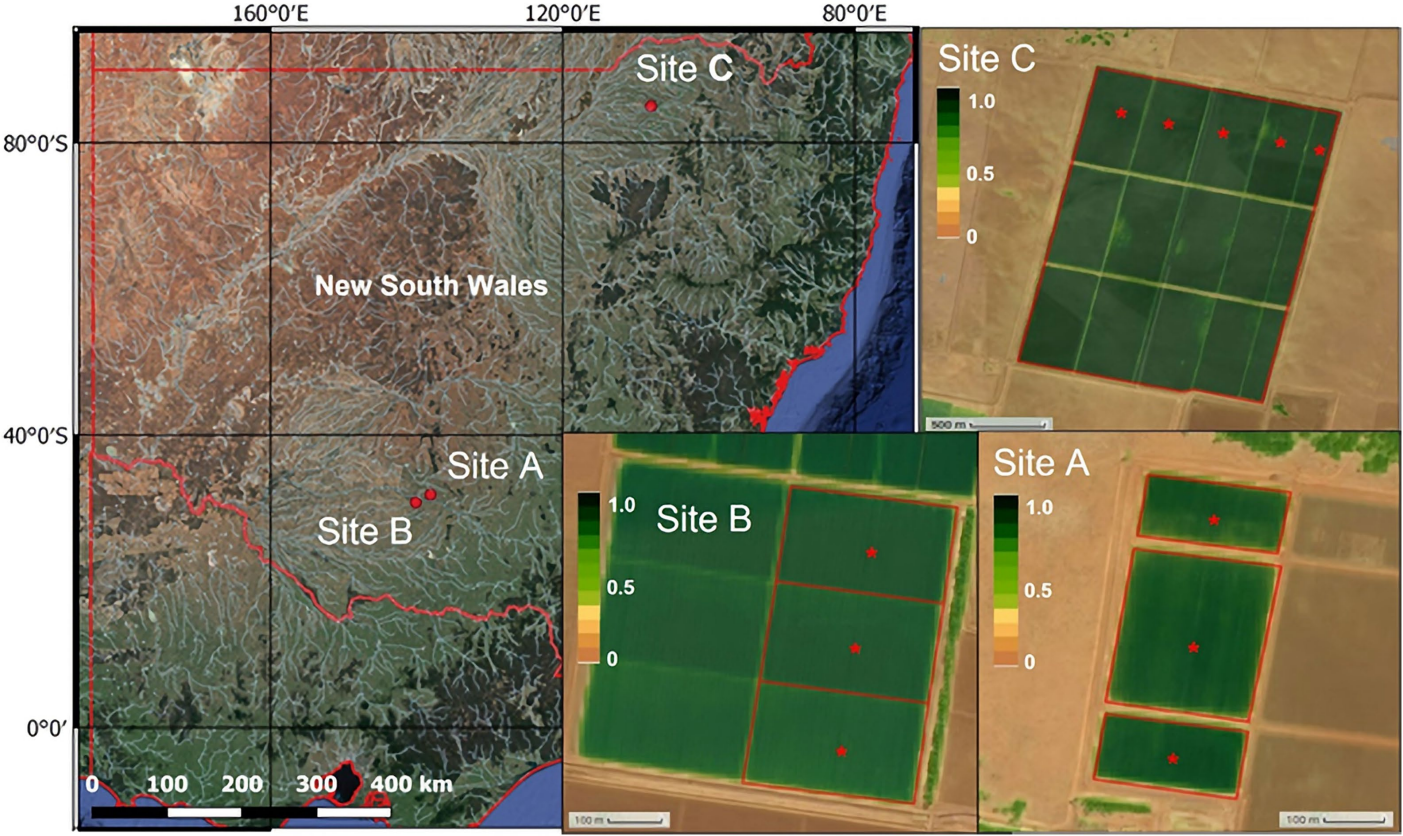
Location of the farms in the Murrumbidgee Valley (sites A and B) and Moree Plains Shire (site C) in NSW, Australia, where soil matric potential was monitored for this study. The red asterisks shown in each NDVI (normalized difference vegetation index) map of each site indicate the monitoring stations. Each station was composed of two matric potential sensors and one temperature sensor buried at 0.20 m depth connected to a WiField datalogger.

The study was conducted during two cotton-growing seasons (2019/20 and 2020/21) with data collected from three commercial irrigated cotton farms located in the Murrumbidgee Valley (sites A and B) and Moree Plains Shire (site C) in the south and north of NSW, Australia, respectively (Figure 1). During a typical cotton growing season, these farms have approximately 500–1,500 irrigated hectares depending on irrigation allocations. Irrigation fields typically have bays ranging from 8–30 ha, so hundreds of irrigated bays may need to be managed for irrigation water applications during the irrigation season.

In the 2019/20 growing season, soil matric potential was monitored at sites A and B, while in the 2020/21 season, it was monitored at sites A and C. The Murrumbidgee area (sites A and B) climate is semi-arid, while it is humid subtropical in Moree in the north of NSW. The weather conditions differed between growing seasons and between cotton producing areas. Total rainfall and reference evapotranspiration (ET_o_) for sites A and B in the 2019/20 cotton growing season (from mid-October to April) were 152 and 1,161 mm, respectively. In the 2020/21 growing season, total rainfall and ET_o_ were 305 and 1,224 mm at site A and 582 and 1,245 mm, respectively, at site C.

Three bays were monitored at sites A (14.6 ha in total) and B (21.0 ha), while five bays were monitored at site C (172.8 ha) in the Moree Plains cotton-producing region where the standard practice is to produce in larger bays (up to ~38 ha in farm C). In all sites, cotton was furrow irrigated employing a bank-less channel irrigation system (Grabham, 2012). Irrigation was scheduled based on the farm consultants’ decision except for site A during the 2020/21 season, where irrigation was triggered based on soil matric potential thresholds and recommendations obtained from the cloud-based IRRISENS platform (Filev Maia et al., 2020). At this site, the grower was notified by text message when soil matric potential at 0.20 m depth at any of the three bays monitored was lower than −40 kPa to be able to order water to the irrigation company and organize the irrigation event 48 h in advance.

### Measured and estimated parameters

Soil matric potential and crop evapotranspiration (ET_c_) were measured and estimated at each site to explore their relationship between irrigation events over the growing seasons. These parameters, rainfall and the growing degree days (GDD), were used as inputs in the machine learning models described in the following subsection.

A WiField logger (Brinkhoff et al., 2017) with two watermark sensors (Model 200SS, Irrometer Company Inc., CA, United States) and one 1-wire temperature shielded sensor (model DS18B20) was used to continuously monitor soil matric potential at each bay. Watermark sensors were installed at 0.20 m below ground as in Kang et al. (2012) and Ballester et al. (2021), where soil matric potential measurements are essential to trigger irrigation events (Brinkhoff et al., 2017). Soil matric potential was calculated using the resistance of each watermark and the soil temperature based on the following equations (Irrometer, 2021):


$$\left\{ \begin{array}{l} st=0,\;r<550\Omega \\ st=\left[\left(r*10^{-3}\right)*23.156-12.736\right]-\left[1+18*10^{-3}*\left(t-24\right)\right],\\ 550\Omega \leq r<1k\Omega \\ st=\frac{-3.213*\left(r*10^{-3}\right)-4.093}{1-0.009733*\left(r*10^{-3}\right)-0.01205*t},\\ 1k\Omega \leq r<8k\Omega \end{array}\right.$$

**Figure 2:**
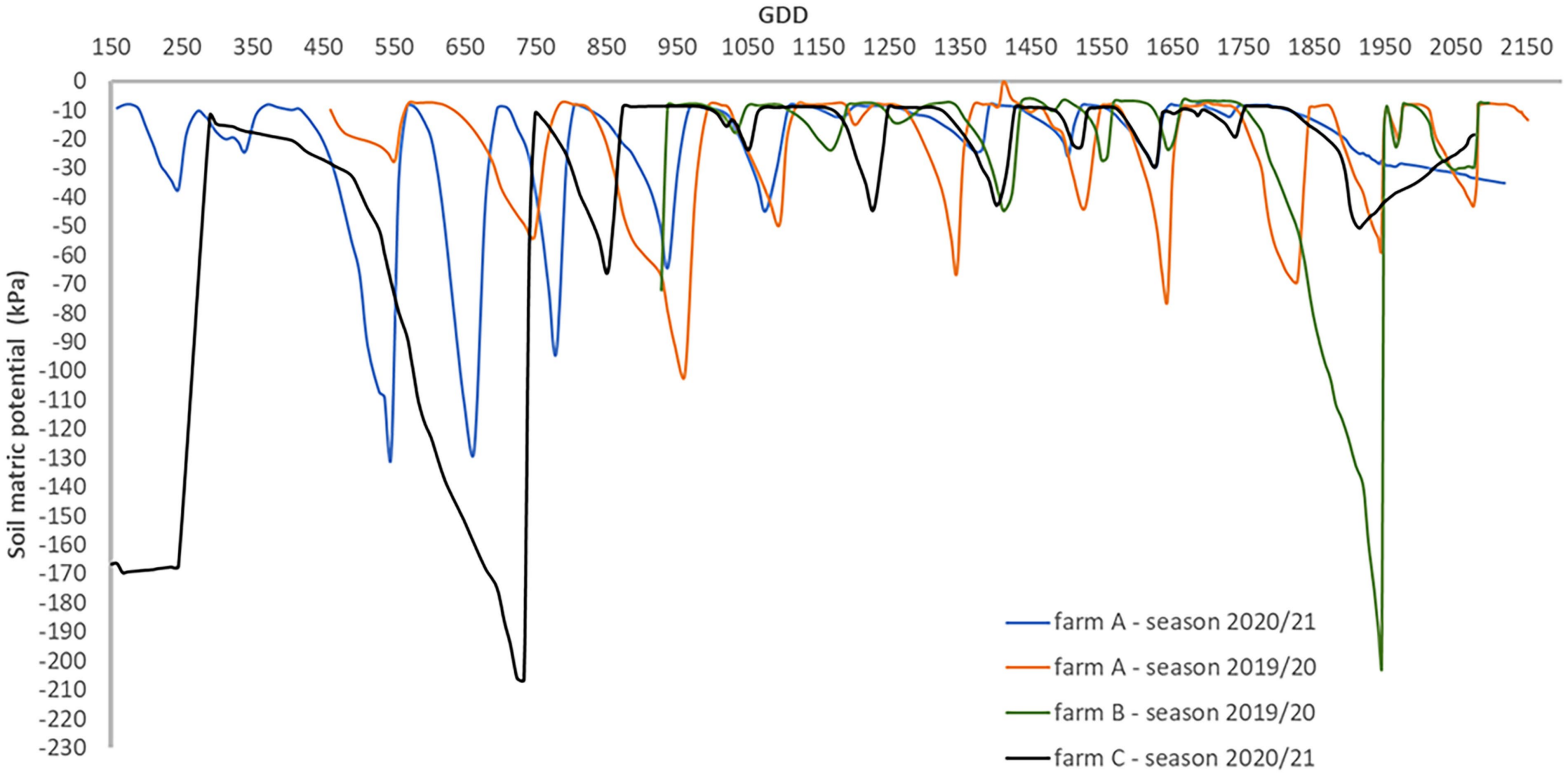
Seasonal soil matric potential evolution measured at 0.20 m depth for each farm and cotton growing season.

**Figure 3:**
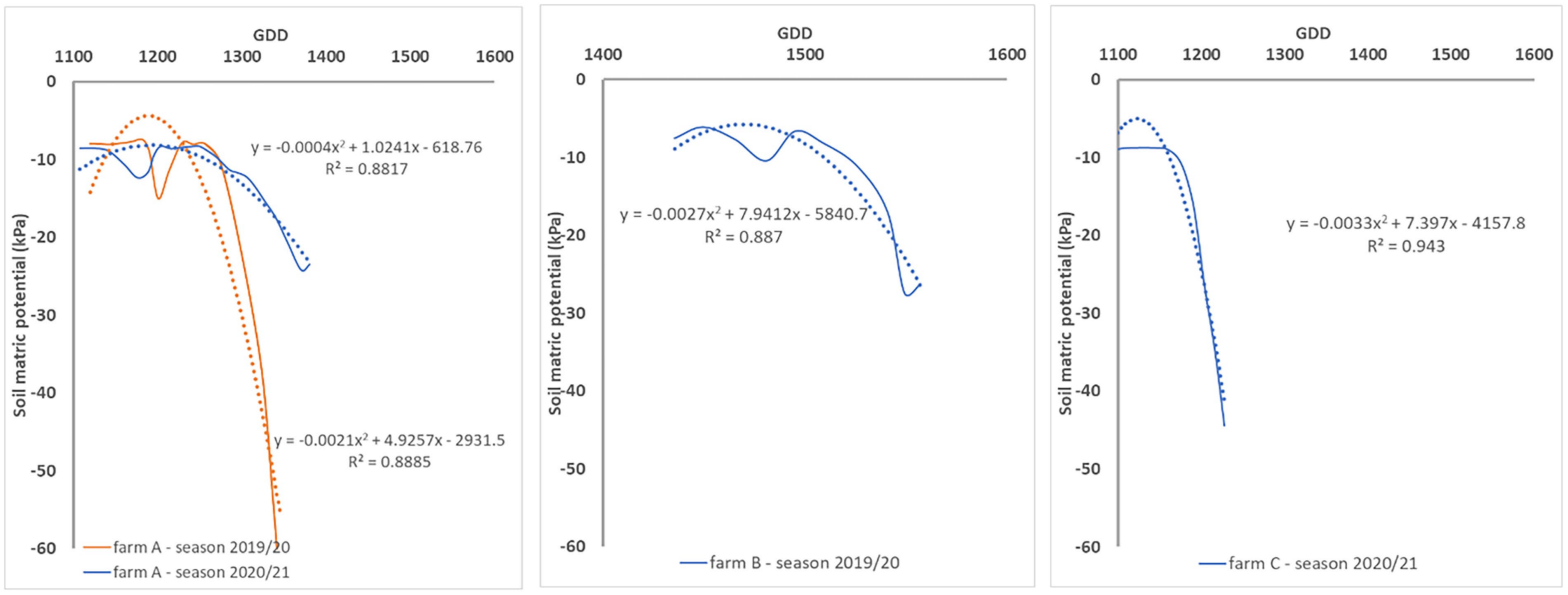
Illustration of the soil matric potential evolution between irrigation events at each farm for different crop phenological stages. The least square function and coefficient of determination (*R*^2^) is shown for each irrigation event.

Figure 2 presents the seasonal evolution of the soil matric potential for each season and site. When soil matric potential readings are observed with more detail between irrigation events, there is an inflexion point that indicates the transition from a saturated (soil matric potential ≥ −10 kPa) to a non-saturated sate (Figure 3). Thus, soil matric potential evolution between irrigation events can be modelled by a second-degree function. The second-degree equation coefficients indicate that the soil matric potential has approximately a linear behavior after the inflexion point. The same behavior was observed at different moments in the season (different crop phenological stages) with weather conditions influencing the soil matric potential decrease rate.

The ET_c_ was estimated following the FAO56 approach (Allen et al., 1998), in which water requirements are obtained as the product between reference evapotranspiration (ET_o_) and a crop coefficient (ET_c_ = ET_o_ × K_c_). ET_o_ (using alfalfa as the reference crop) was estimated by the Penman-Monteith equation using weather information obtained from the nearest meteorological stations to each site. The crop coefficient was estimated from satellite-based NDVI images using the relationship reported in Trout and Johnson (2007) (*K*_c_ = 1.37 NDVI – 0.086). NDVI images of each site were obtained using the Google Earth Engine API to access the Sentinel-2 top of atmosphere reflectance data collection. The extracted images were processed to eliminate those with more than 5% of pixels with clouds. The remaining images were used to calculate the NDVI according to. As Sentinel-2 satellites do not provide daily data in the monitored areas, NDVI was estimated using linear regression for those dates with no images available.

Daily ET_c_ was used to calculate the cumulative evapotranspiration between irrigation events as follows:


$$ET_{cn}=\overset{n}{\underset{d=0}{\sum }}ET_{c}\left(d\right)$$

where *d* is days since the last irrigation, its value is zero on the day that irrigation occurs, and maximum *n* on the day before the next irrigation. The cumulative ET_c_ has the same cumulative behaviour as the soil matric potential.

The GDD index was another input parameter used in the models to account for the effect of temperature on crop development in each cotton-producing region (McMahon and Low, 1972). This index is computed from daily maximum and minimum air temperature data and a temperature threshold (the base temperature) at which crop growth stops. That for cotton is 12°C as follows:




$$GDD=\overset{n}{\underset{k=0}{\sum }}\frac{\left(Tmax_{k}-12)+(Tmin_{k}\right)}{2}$$

### Machine learning proposed approach

#### Data selection and pre-processing

In all monitored sites there were three sources of data: WiField loggers, Sentinel-2 data collections, and weather stations installed in each site or close to it. Table 2 depicts the machine learning input data collected in the field. Data cleaning and organization were the same in all seasons according to the following criteria:

- soil matric potential readings range between 0 kPa and –200 kPa – readings lower than this value do not represent correct values;
- soil matric potential at each bay was the average of two sensors;
- weather data was collected from on-site weather stations (sites A and C) or nearby (site B);
- remote sensing data extraction occurred every 5 days when Sentinel-2 covered the farm area at least once;
- each satellite image and data extraction considered only points from the polygon representing each bay of the evaluated paddock, and the cloud coverage in such polygon must be less than 5% of the pixels in the image.

**Table 2:** Potential input features used in this study to estimate soil matric potential.

Features	Source	Dimension/frequency
Solar radiation	Weather station	Daily value
GDD	Weather station	Daily value
NDVI	Remote sensing	Daily estimated
ETc_n_	calculated	Cumulative evapotranspiration between irrigation events (daily)
Rain_n_	Weather station	Cumulative rainfall in mm between irrigation events (daily)
Soil matric potential	Sensor/estimated	Daily average

GDD, growing degree days; NDVI, normalized different vegetation index; ET_c_, cumulative crop evapotranspiration.

All data were organized sequentially according to the GDD to enable comparisons between crops from different locations and seasons. Two datasets were created to evaluate the algorithms. One dataset included data for each site and growing season individually, and the other dataset was composed of data from sites and seasons.

The time interval of data used to train and evaluate the algorithms was from the beginning of the monitoring period, excluding the initial 24 h after installing the sensors (wet conditions) and data for GDD < 600 (plants not emerged yet or emerging), up to 2,200 GDD. No irrigation events were undertaken in the evaluated paddocks after 1,700 GDD. The selection of the parameter set used as algorithms inputs was based on two criteria: (i) the training response (lower errors), and (ii) one parameter cannot be a linear combination of the other input parameters. This last condition forbids having NDVI and ETc in the same input set. The selected input set for all evaluations was composed of GDD, ETc_n_ and Rain_n_. The GDD index is relevant to locating the input data in the proper phenological crop stage. The other two parameters are essential to evaluate the soil water availability.

#### Machine learning algorithms

The machine learning models must learn the relation kPa/mm and forecast the soil matric potential in non-monitored areas giving one set of input parameters. As this relation is nonlinear, two models were selected to deal with this nonlinearity. First, the Support Vector Regression (SVR) algorithm is based on the Support Vector model to estimate the value of a point as the best hyperplane that represents the given sample (Smola and Schölkopf, 2004). The second algorithm was a Dense Multilayer Perceptron (DMP) neural network that can be understood as an ANN in which one neuron is connected with all neurons from the subsequent layers (Arnold et al., 2019). The SVR algorithm was implemented based on the Sklearn package, and the DMP model was developed using the Tensorflow API (Abadi et al., 2016).

Table 3 presents the configuration of each algorithm. All tests were performed using the k-fold method (Bengio and Grandvalet, 2004), which uses a cross-validation technique when the dataset is split in *k* folds with approximately the same number of samples. The algorithms are tested *k* times, each one changing the validation subset, i.e., each interaction deals with a selection of the *k* – 1 folds to training and one to test training, and each fold is used to test the algorithm once. The average of tested folds determines the algorithm’s accuracy.

**Table 3:** Description of the machine learning models configuration.

Model	Layers/kernel	Optimizer
DMP	Input: 1 × 3 2 dense layers 32 neurons each – Tanh Output 1 × 1	Adam
SVR	Input: 1 × 3 Radial basis function (RBF) kernel Output 1 × 1	–

DMP, dense multilayer perceptron; SVR, support vector regression.

Before splitting data in folds, they were shuffled to mix the order of the points in the farm dataset and to mix data from different farms and seasons in the second type of dataset. In the last type of dataset, this is a way to avoid points from just one farm being part of a fold and data from other farms in the other fold. The risk of not doing this shuffle is to make the algorithms, particularly DMP, learn the relation kPa/mm related to one grower’s practice instead of a pattern in the cotton crop.

The SVR and DMP models received the same training inputs format 1 × 3, i.e., one input has three parameters, and present the result as 1 × 1, i.e., one output with one estimated/forecasted point. The input set was a combination of parameters from Table 2, and the output was soil matric potential in all evaluations.

The *R*^2^ (coefficient of determination) obtained for each tested fold was considered to evaluate the algorithms’ training and responses. However, as the objective of the algorithm evaluation is not to estimate the exact soil matric potential but to provide an admissible value, the algorithms were also evaluated based on the percentage of estimates within an interval of 10 kPa. This interval was selected because of the fluctuation observed in soil matric potential between sensors installed in the same bay next to each other when soil matric potential data for all the sites was assessed.

## Results

### Relation between soil matric potential and ETc (kPa/mm)

**Figure 4:**
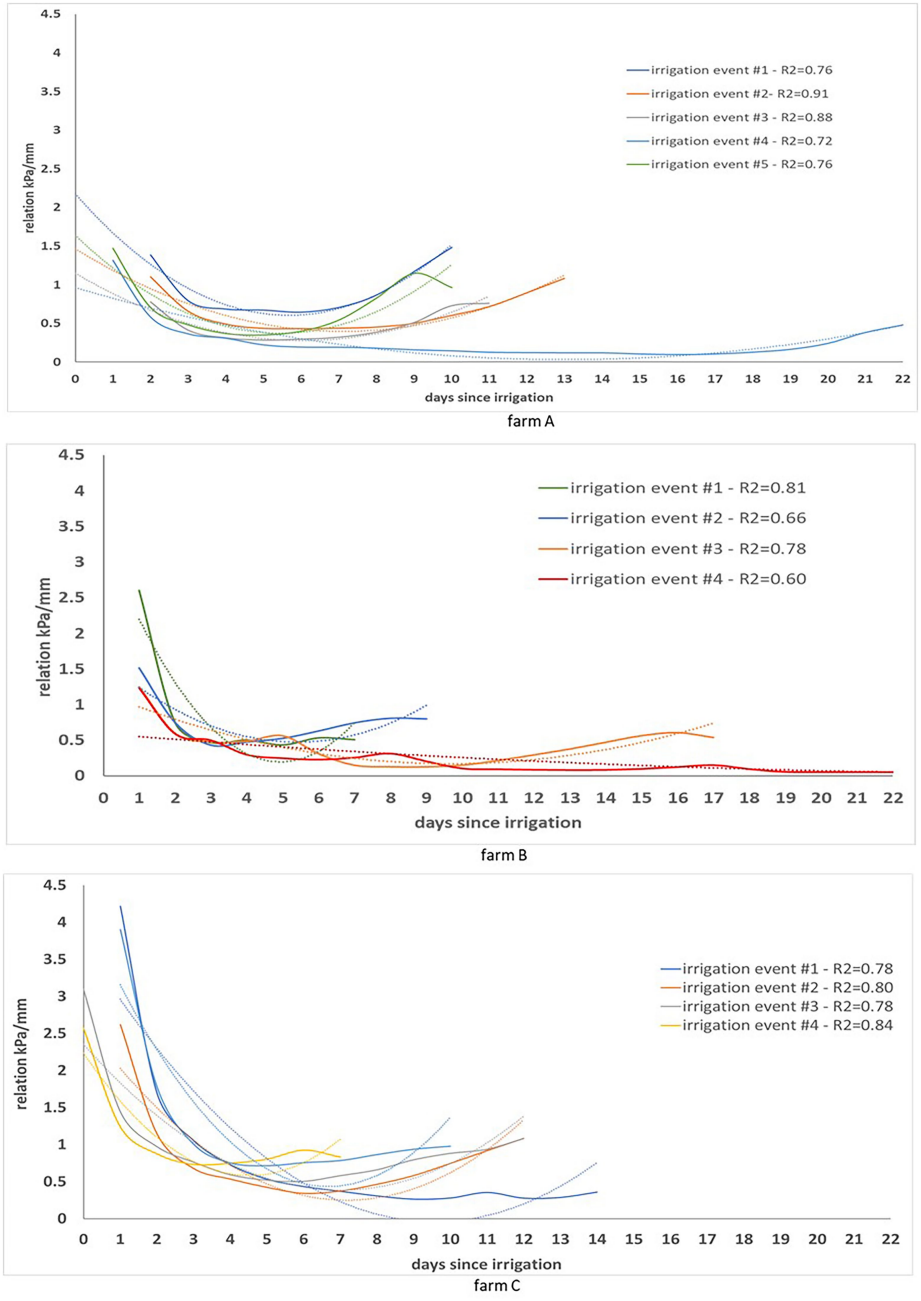
Evolution of the relation kPa/mm observed during several irrigation events at each farm. Within each farm, the least square function and coefficient of determination (*R*^2^) is shown for each irrigation event.

The relation between soil matric potential and cumulative evapotranspiration between irrigations expressed in kPa/mm, represents the ratio of the soil matric potential and water demand according to the crop development. This relation can be expressed as a second-degree function (Figure 4) in which the inflexion point corresponds to the inflexion point from the soil matric potential chart (Figure 3). It allows the observation of the dynamics of soil matric potential even when soil is saturated, and the crop water demand cannot be readily evaluated in the soil matric potential chart. Figure 4 shows the relationship between soil matric potential and cumulative evapotranspiration in several irrigation events (one line for irrigation event). The *R*^2^ of each relation kPa/mm is similar in farms A and C, even though irrigation management was different in each farm. In farm A irrigation scheduling was done based on the recommendations of an automatic irrigation control system while in farm A and B it was done based on the water managers’ practices. The relation was not affected by weather events or the crop phenological stage. The same relation was observed at different phenological crop stages (see Figure 4 – farm B – irrigation event #4 in the end of the growing season when crop water demand is lower, and irrigation is not needed) and weather conditions. However, rainfall had a decreasing effect on the slope of such relation as can be observed in Figure 4 (farms A and B– irrigation event #4) when it remained flat for longer.

In farms A and B, the relation kPa/mm had a maximum value close to 2.5, while in farm C the maximum value was close to 4.5. The difference could be related to the soil and weather conditions that were different for farms A and B compared to farm C. Due to the difficulty of having an analytical model that encompasses such relation, the alternative is to apply a machine learning model. Such models may learn the relation kPa/mm and predict the soil matric potential through remote sensing data in areas not monitored by sensors.

### Algorithms’ evaluation in individual farms

The algorithms’ evaluation in individual farms comprises the automatic irrigation in farm A and non-automatic (or traditional) irrigation strategies in farms B and C. Farms A and B present a smaller monitored area than farm C. Consequently, there are fewer points in farms A and B compared to farm C to train and evaluate resultant model from each algorithm. Due to this situation, farms A and B had only three folds to be evaluated, while farm C had five folds. Therefore, three folds from each farm are presented to evaluate the algorithms’ responses in all following analyses.

**Figure 5:**
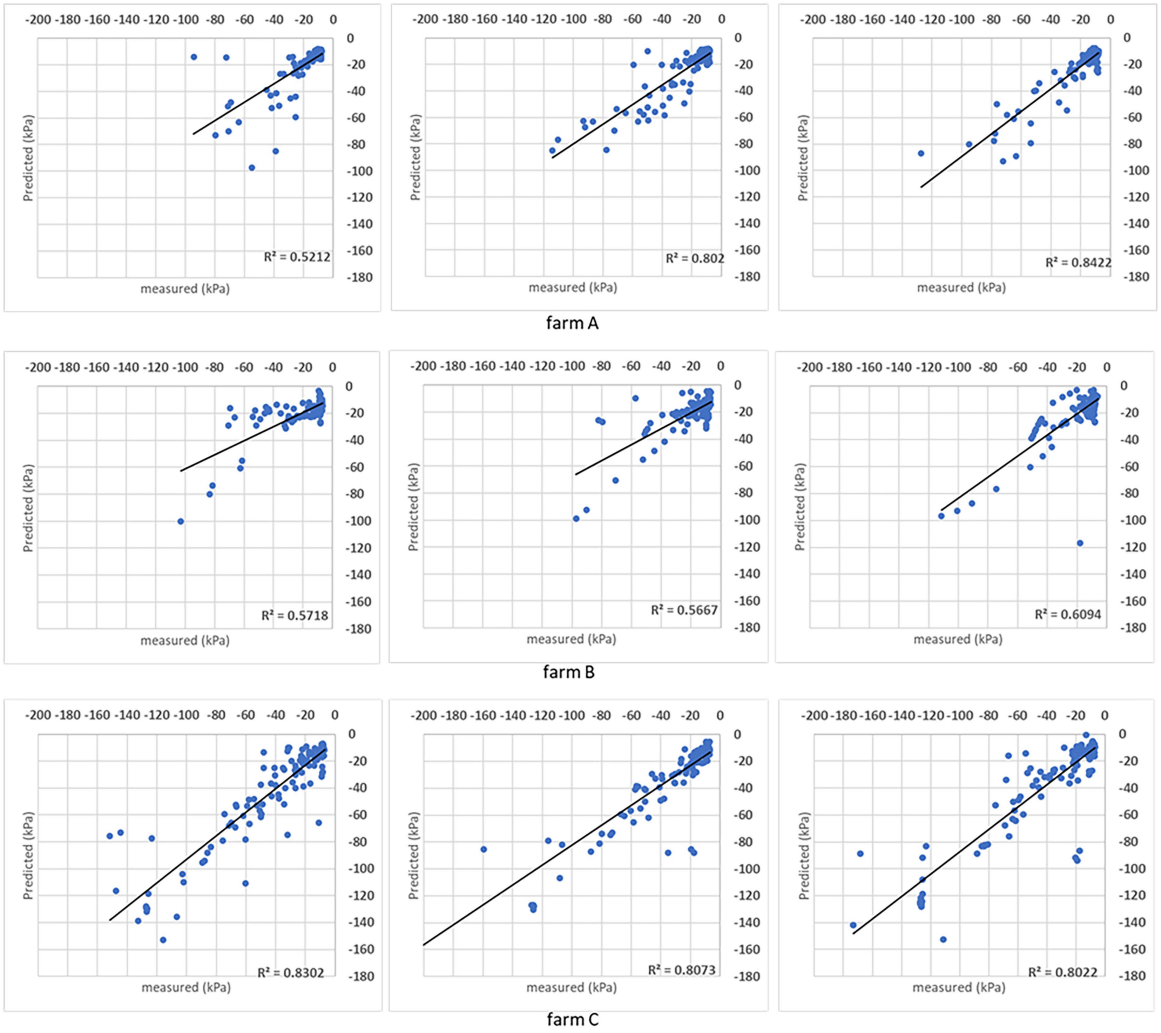
Results obtained with the DMP model when the analysis was done for individual sites. For each farm, the coefficient of determination (*R*^2^) obtained for the comparison between measured and predicted soil matric potential is shown for three folds.

**Figure 6:**
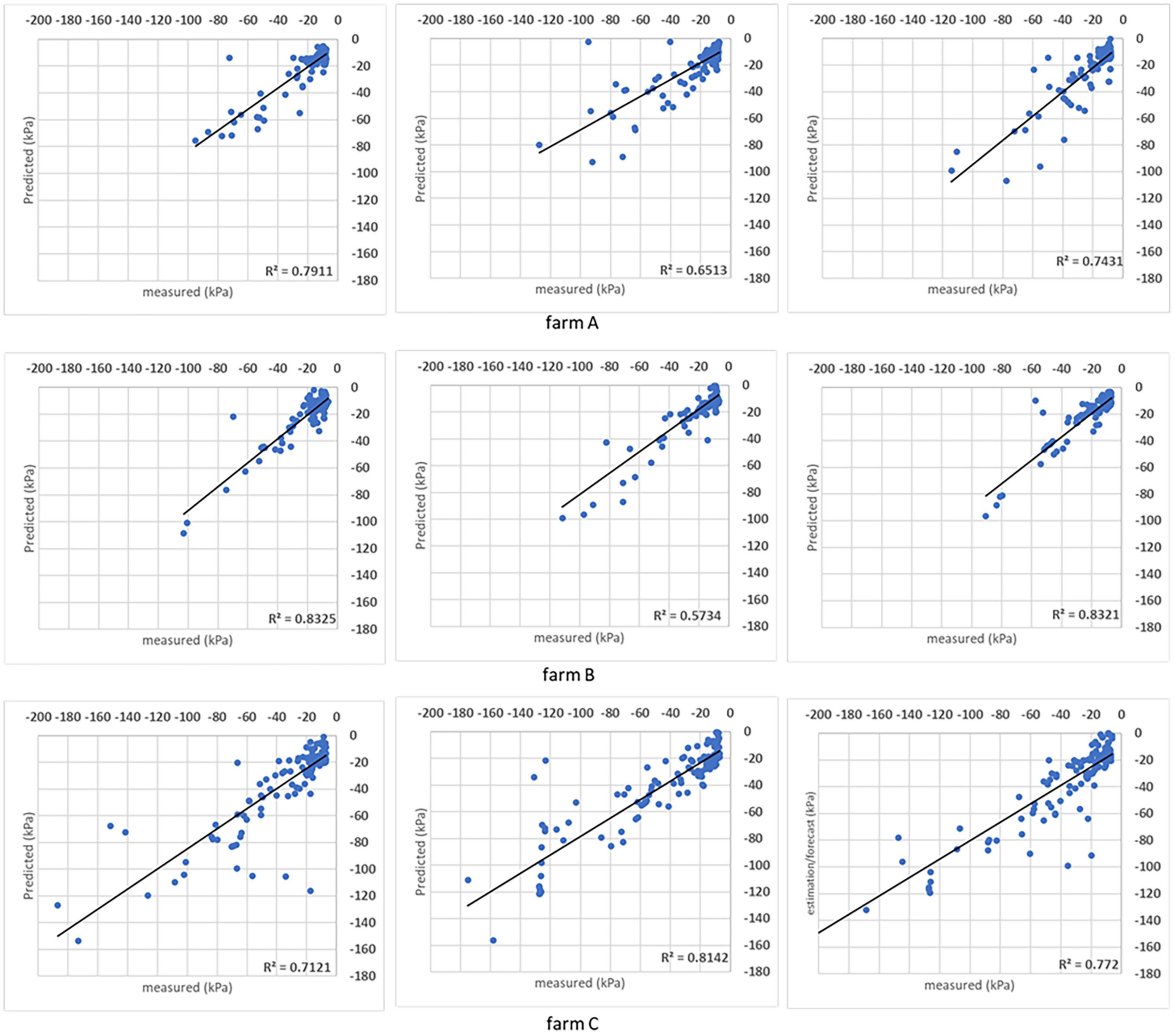
Results obtained with the SVR model when the analysis was done for individual sites. For each farm, the coefficient of determination (*R*^2^) obtained for the comparison between measured and predicted soil matric potential is shown for three folds.

The DMP presents R^2^ above 0.80 in farms A and C (Figure 5), indicating the automation of the irrigation process did not play a decisive role in the algorithm estimation capability, which can be confirmed by SVR results (Figure 6). That SVR algorithm presents inferior performance compared to DMP in farms A and C, but the opposite in farm B, when the performance was substantially superior to DMP. It is possible to evaluate a sequence of points similar to a line in farm B (second and third folds in Figure 5) not closed from the *R*^2^ line estimated by the DMP algorithm.

**Figure 7:**
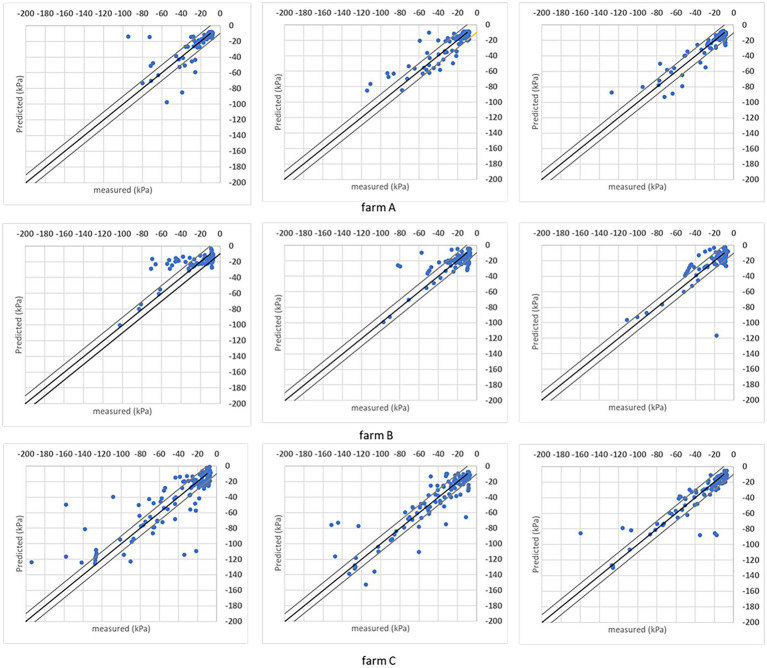
Results obtained with the DMP model when the analysis was done for individual sites considering the *R*^2^ = 1 (black line) and ± 10 kPa interval between the grey lines. Three folds are shown for each farm.

**Figure 8:**
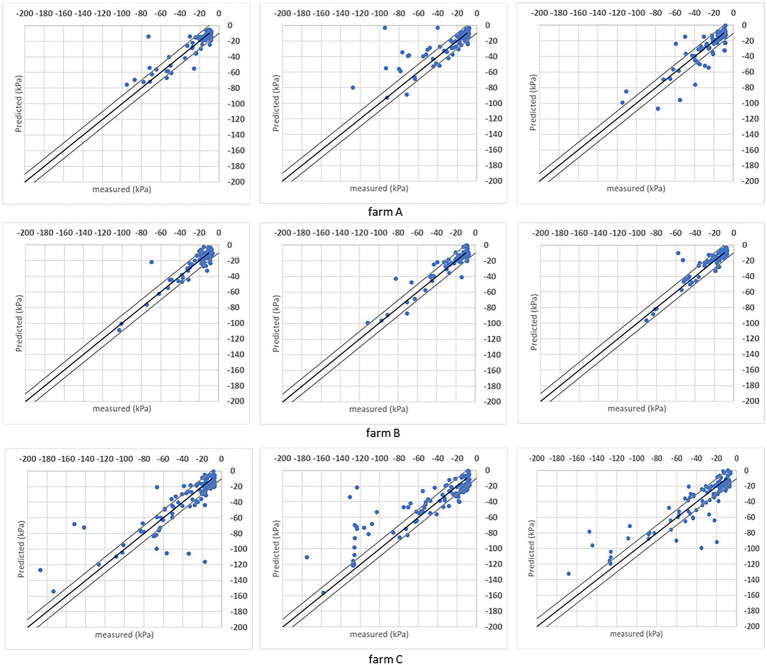
Results obtained with the SVR model when the analysis was done for individual sites considering the *R*^2^ = 1 (black line) and ± 10 kPa interval between the grey lines. Three folds are shown for each farm.

It is also evaluated the soil matric potential estimations/predictions versus measured points distribution in a ±10 kPa interval. Charts in Figures 7, 8 have the *R*^2^ = 1 (black line) and the ±10 kPa represented between grey lines. Both charts represent individual farms with three folds each.

Both models’ estimations below –50 kPa present equivalent results, while DMP presents better estimations for points below –50 kPa in farms A and C. The percentage of estimated points in the ±10 kPa interval; across all farms showed a satisfactory performance (Table 4). The DMP had better performance than SVR in farm C, while there is no difference between algorithms in farm A. The monitored area in farm C is more extensive than on farms A and B. Due to the size of the bays in farm C, an irregular moisture distribution in the bays could be observed on the same day, which means the algorithms had to deal with significant differences in soil matric potential corresponding to the same GDD values in the input parameters.

**Table 4:** Percentage of estimated points between the ±10 kPa and ±15 kPa intervals in the analysis for all farms and seasons combined applying the DMP and SVR models.

Fold#	Points in ±10 kPa band		Points in ±15 kPa band	
	DMP	SVR	DMP	SVR
1	0.67	0.77	0.76	0.83
2	0.65	0.68	0.78	0.76
3	0.71	0.72	0.81	0.82
4	0.75	0.74	0.83	0.83
5	0.69	0.74	0.82	0.83
Avg.	0.69	0.73	0.80	0.82

DMP, dense multilayer perceptron; SVR, support vector regression.

Farm A presents the same results for both algorithms reflecting the regularity promoted by the automation process in the irrigated areas. However, farm B presented a significant difference between algorithms with SVR superior to DMP, getting 90% of corrected estimated points in the ±10 kPa band (see farm B – SVR dispersion in Figure 8).

### Evaluation of algorithms considering all farms and growing seasons combined

In individual farm evaluation, the differences in irrigation strategies did not cause overfitting in the neural network model but could cause a bias in the results (grower practices). Combining the measured points from all farms and seasons and shuffling the points are essential to creating the evaluation dataset when such bias is not present.

**Figure 9:**
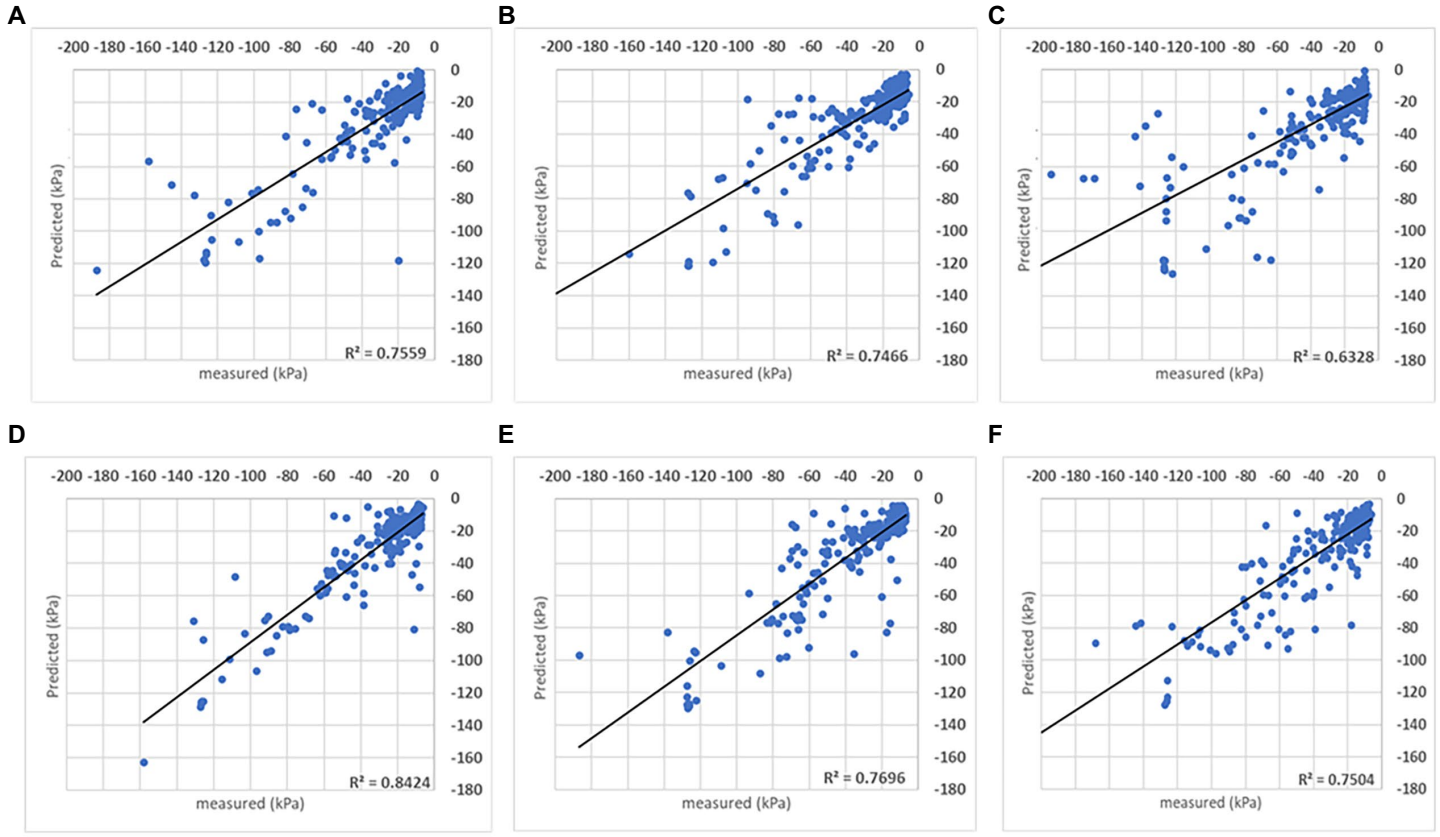
Results obtained with the SVR **(A–C)** and DMP **(D–F)** algorithms when the analysis was done combining data from all sites and seasons. The coefficient of determination (*R*^2^) obtained for the comparison between measured and predicted soil matric potential is shown for three folds.

Figure 9 presents the soil matric potential measured versus estimated to evaluate the responses provided by SVR and DMP. Both algorithms had equivalent results in all testing folds. According to the *R*^2^ metric, both algorithms present similar learning capabilities. The DMP reached *R*^2^ = 0.8424 in the fold (d), and SVR reached *R*^2^ = 0.7559 in the fold (a), meaning the DMP model provided a more accurate estimation of the soil matric potential in the root zone with a given input set.

**Figure 10:**
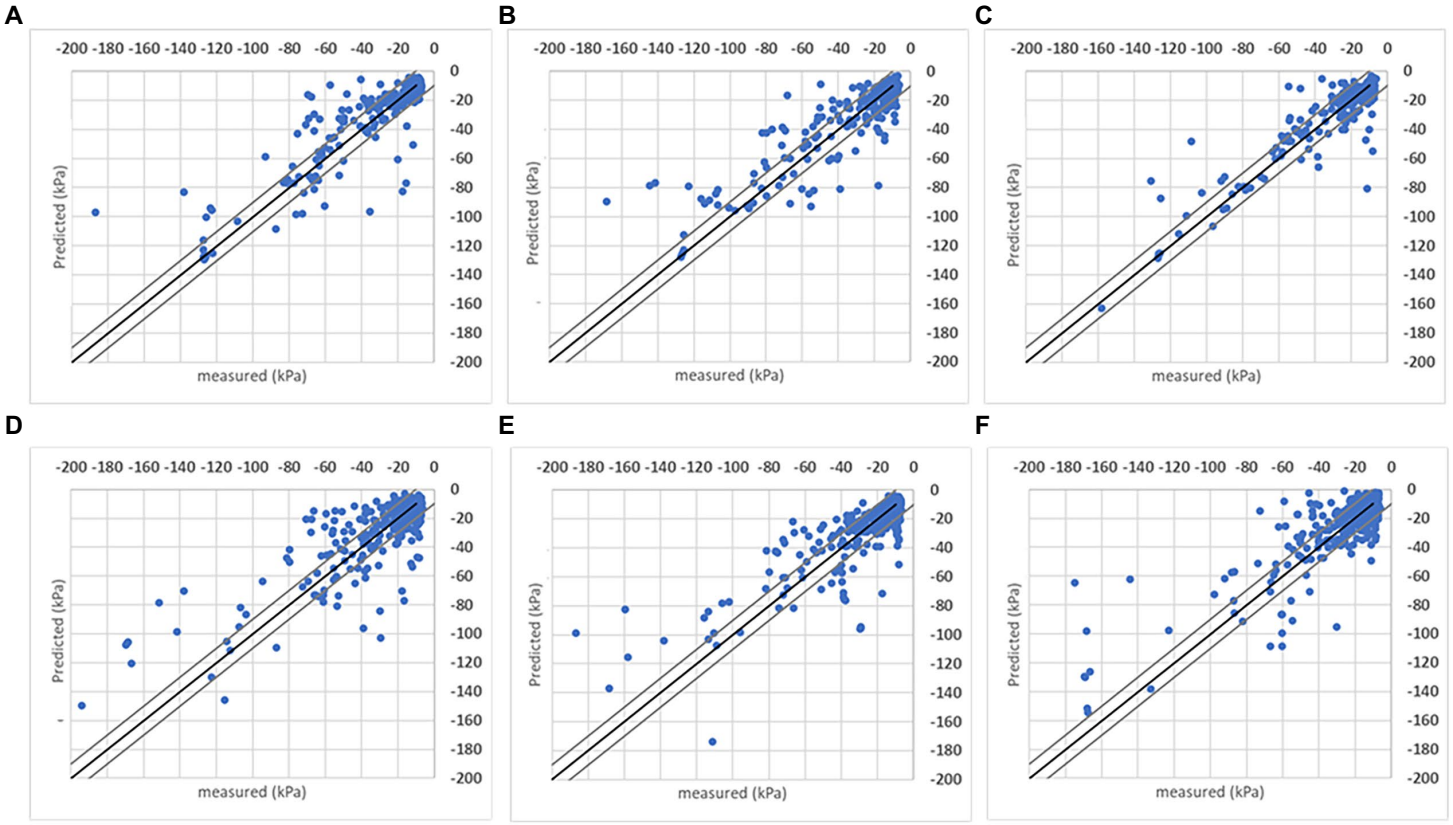
Results obtained with the SVR **(A–C)** and DMP **(D–F)** algorithms when the analysis was done combining data from all sites and seasons considering the *R*^2^ = 1 (black line) and ± 10 kPa interval between the grey lines. Three folds are shown for each farm.

The performance of each model considering the ±10 kPa interval is represented in Figure 10. Data organization followed the k-fold method that generated other sets of folds for training/evaluation sessions. With the reorganization of points, the same characteristics were found and represented in Figure 9 (compared to Figure 8). Both models present similar responses when estimated points are contained in the proposed interval, reflecting the field’s measurements. Evaluating how many estimated points are in the ±10 kPa (Table 4), the SVR presents slightly better results than DMP. On average, 69.56% of points in DMP and 72.98% of points in SVR are in the proposed interval. Increasing the interval to ±15 kPa, both models present the same results (~80%) since the interval includes more correctly predicted points below –60 kPa.

## Discussion

### Relationship between soil matric potential and ET_cn_

Soil water dynamics are influenced by many bio-physical factors forming a complex system that is difficult to capture by analytical models without a significant number of input parameters and a complete understanding of their relationship. However, changes in soil moisture storage can be determined by a soil water balance model as the difference between water added to the soil (precipitation and irrigation) and water lost in the system (deep percolation, run off, lateral flow and evapotranspiration; O’Geen, 2013). In the context of this study, decreases in soil moisture storage in the top 0.20 m of soil are mainly due to the crop evapotranspiration. The relationship between soil matric potential and cumulative crop evapotranspiration between irrigation events (kPa/mm) that is expressed by a second-degree function should then represent this system. The *R*^2^ obtained for this relationship was in most cases >0.72 even though data was collected at different times in the season and from farms with different soil characteristics, water management strategies and weather conditions (Figure 4). For example, in farms A and B, the 2019/20 cotton growing season was hotter and dryer than the 2020/21 growing season. The highest (47°C) and lowest (19°C) maximum temperatures for the month of January since 1960 were recorded in the 2019/20 and 2020/21 growing seasons, respectively. These results suggest that the soil matric potential-ET_cn_ relation between irrigation events is stable and machine learning models that capture this relationship can potentially be used to estimate soil matric potential from ET_c_ measurements.

### Performance of the models for individual and combined datasets

The SVR and DMP models were used in this study to learn the pattern of the proposed relationship to be able to indirectly estimate soil matric potential in areas not monitored with sensors from satellite-NDVI derived ET_c_ measurements. This was possible without using specific soil characteristics as data input as it was required in other studies such as Villani et al. (2018). The approach followed in this work is different from other studies using remote sensed data as an input parameter in machine learning models for the estimation or prediction of soil moisture (Ahmad et al., 2010; Coopersmith et al., 2016; Torres-Rua et al., 2016; Sungmin and Rene, 2021). In Ahmad et al. (2010), microwave backscatter observations and incidence angle from Tropical Rainfall Measuring Mission (TRMM), NDVI and simulated soil moisture data (at 0.10 m depth) were used as input parameters in a Support Vector Machine model to estimate soil moisture with good results. In Torres-Rua et al. (2016), surface soil moisture was estimated at spatial and temporal resolution using a Relevance Vector Machine model by combining *in situ* soil moisture and weather data with satellite-derived evapotranspiration (METRIC model). These models used in Ahmad et al. (2010) and Torres-Rua et al. (2016), were effective in estimating volumetric water content in the top 0.05 m of soil but its feasibility for estimating soil moisture at deeper soil layers was not studied which limits the applicability of these models to irrigation decision support systems. In the study here presented, soil matric potential was preferred to soil water content because of its suitability for irrigation automation and unneeded soil-specific calibration. The SVR and DMP models learnt the pattern of the soil matric potential and crop evapotranspiration relationship using the GDD index, satellite NDVI-derived ET_c_ and rainfall as input parameters. The models’ output was the estimated soil matric potential at 0.20 m depth, where thresholds can be used in practice to trigger irrigation events. Gumiere et al. (2020) and Dubois et al. (2021) also explored machine learning models for predicting soil matric potential in cranberry and potato crops, respectively, for irrigation management. In Gumiere et al. (2020), a Random Forest (RF) model with rainfall, reference evapotranspiration and soil matric potential measurements at 0.10 m as input data predicted hourly soil matric potential with an *R*^2^ of 0.58. The average *R*^2^ obtained with the DMP and SVR models for individuals farms and for the entire dataset combined was ≥0.70 in this study. The best performance of the models here tested could be related with the fact that Gumiere et al. (2020) predicted soil matric potential hourly while daily values were estimated in this work. The RF, SVR, and Neural Network (NN) models used in Dubois et al. (2021) had a higher performance (*R*^2^ > 0.92) in predicting soil matric potential than the models used here although remotely sensed data was not used as an input parameter in their study and thus it cannot be used for the purpose proposed in this study.

### Soil moisture estimation by machine learning models

Both SVR and DMP models performed well in estimating soil matric potential (Tables 4, 5). This is a strong indication that the kPa/mm relation can be used to estimate the soil matric potential in the root zone of non-monitored areas from NDVI data, rainfall, and the GDD index that represents the development stage of the crop. The performance assessment for individual datasets (farms) showed that both models performed similarly and that they performed slightly better in farm A and B than in farm C. In particular, the SVR model in farm B presented the highest accuracy, but this could just be that the models fit better the dataset for those particular farm and season. The performance of the models considering the ±10 kPa interval when data from all sites and seasons was combined was slightly worse than for individual farms although still with an accuracy around 70% (Table 4). The SVR model estimated 4% more readings within the ±10 kPa interval than DMP. The performance improved significantly in both models when the interval increased to ±15 kPa mainly due to the inclusion of estimates of soil matric potential between −60 kPa and −120 kPa within this larger interval. The dataset used for the training and testing of the models had more readings between 0 and −60 kPa than below this value because irrigation was scheduled in these sites to ensure plant water availability and avoid water stress conditions. Consequently, the soil matric potential dataset below −60 kPa was scarce for a proper training of the models and the accuracy in estimating soil matric potential in drier soil was lower.

**Table 5:** Percentage of estimated points between the ±10 kPa interval in the analysis for individual farms with the DMP and SVR models.

Fold#	Farm A		Farm B		Farm C	
	DMP	SVR	DMP	SVR	DMP	SVR
1	0.89	0.88	0.75	0.94	0.55	0.63
2	0.78	0.80	0.77	0.87	0.60	0.61
3	0.83	0.82	0.78	0.90	0.78	0.64
4	–	–	–	–	0.80	0.57
5	–	–	–	–	0.84	0.58
Avg.	0.84	0.84	0.77	0.90	0.72	0.61

DMP, dense multilayer perceptron; SVR, support vector regression.

## Conclusion

This study showed that soil matric potential and cumulative ET_c_ between irrigation events (kPa/mm) have a stable and robust relationship that integrates the effects of soil type and weather condition in a second-degree function. It also demonstrated that machine learning models with capability to process time-dependent parameters such as the DMP and SVR applied here can learn the patter of the soil matric potential-ET_cn_ relation. This offers the possibility of accurately estimating soil matric potential in the root zone of crops in non-monitored areas with in-filed sensors from remotely sensed ET_c_ estimates. The approach is scalable to farms with multiple irrigation fields without the limitations of on-ground sensing related to the cost and organization of the sensors network.

The assessment of this relation kPa/mm with machine learning models provides a new technique to estimate soil tension in the root zone of cotton crops although it is potentially suitable for other crops. Future work can consider collecting data across wider areas and more seasons to refine the relation kPa/mm and machine learning models’ performance. Additionally, research on expanding the relation kPa/mm in other crops to evaluate if the relation sustains and how well it represents the complexity of the crop/soil/water/weather system has merit for other industries.

## Data availability statement

The raw data supporting the conclusions of this article will be made available by the authors, without undue reservation.

## Author contributions

JH, CL, and RM: project conceptualization, data evaluation and validation, and review and editing. RM and CL: methodology and writing. RM: relation and machine learning models. JH: funding acquisition. All authors contributed to the article and approved the submitted version.

## Funding

This work is supported by funding from the Australian Government Department of Agriculture, Water and the Environment as part of its R&D for Profit Program and the Cotton Research and Development Corporation.

## Conflict of interest

The authors declare that the research was conducted in the absence of any commercial or financial relationships that could be construed as a potential conflict of interest.

## Publisher’s note

All claims expressed in this article are solely those of the authors and do not necessarily represent those of their affiliated organizations, or those of the publisher, the editors and the reviewers. Any product that may be evaluated in this article, or claim that may be made by its manufacturer, is not guaranteed or endorsed by the publisher.
